# Experience in neuromuscular diseases in children and adolescents and their comorbidities in a tertiary hospital

**DOI:** 10.1186/s13052-021-01176-4

**Published:** 2021-11-16

**Authors:** J. A. Gascón-Navarro, M. J. De La Torre-Aguilar, J. A. Fernández-Ramos, J. Torres-Borrego, J. L. Pérez-Navero

**Affiliations:** 1 Department of Pediatrics. Reina Sofia University Hospital, Córdoba University, Maimónides Biomedical Research Institute of Córdoba (IMIBIC), Avd. Menéndez Pidal s/n. PC, 14004 Córdoba, Spain; 2 Neuropediatrics Unit. Department of Pediatrics. Reina Sofia University Hospital, Córdoba University, Córdoba, Spain; 3 Center for Biomedical Research on Rare Diseases (CIBERER), ISCIII, Madrid, Spain; 4 Pediatric Pneumology Unit, Department of Pediatrics. Reina Sofía University Hospital, Córdoba University, Córdoba, Spain

## Abstract

**Introduction:**

Neuromuscular diseases include a large group of heterogeneous and rare pathologies that affect different components of the motor unit. It is essential to optimize resources to know the prevalence of comorbidities in the most frequent groups to establish an early multidisciplinary approach in a specialized setting.

**Patients and methods:**

Retrospective descriptive study of pediatric and adolescent patients with neuromuscular diseases (NMDs). The Inclusion criteria were NMDs patients with motor neuron involvement divided into three groups, depending on the affected component of the motor unit. Group I: involvement of the motor neuron; Group II: peripheral neuropathies; Group III: myopathies. Demographic variables, association with comorbidities, need for respiratory support, and rehabilitative treatment were collected in each group.

**Results:**

Ninety-six patients who met the inclusion criteria were studied. In group I, when compared to the other two groups, a higher incidence of scoliosis (68.3%, *p* = 0.011), deformity of the rib cage (31.3%, *p* = 0.0001), chronic respiratory insufficiency (62.5%, *p* = 0.001) and bronchial aspiration (12.5%, *p* = 0.03) was detected. In this group, 50%of the patients required non-invasive mechanical ventilation (*p* = 0.0001). The in-hospital requirement for respiratory physiotherapy was higher in group I (75%, *p* = 0.001). We observed a higher incidence of scoliosis in Group III compared to Group II.

**Conclusions:**

Neuromuscular diseases with motor neuron involvement present more comorbidities and require an early approach after diagnosis to improve prognosis.

## Introduction

Neuromuscular diseases (NMDs) comprise a wide range of syndromes, constituting more than 150 pathologies, including motor neuron involvement, peripheral neuropathy, myopathies, and neuromuscular junction disorders [1]. Muscle weakness is a feature shared by all these conditions. A good knowledge of the natural history of each NMDs is essential to guarantee the optimal time for therapeutic interventions and the start of rehabilitation. In some of these syndromes, the presenting manifestation is a functional sign or deformity. The timing of the treatment of spinal deformities is crucial, due to the hypotonia of the paraspinal musculature during growth. A multidisciplinary approach is always required, muscular and respiratory physiotherapy being key.

In NMDs, the musculoskeletal system is affected early, which compromises the acquisition of motor milestones, especially the development of gait and stability [2]. Loss of muscle strength has a negative impact on the skeletal system, producing muscle changes, scoliosis, and thoracic deformities, mainly due to the weakness of the thoracic, abdominal and paraspinal muscles, as well as the diaphragm. For this reason, the incidence of restrictive respiratory insufficiency is frequent, with a decrease in vital capacity, total lung capacity and functional residual capacity, as well as all lung volumes [3, 4]. In evolution, there is a decrease in inspiratory and expiratory force, peak flow, and loss of the ability to cough. This contributes to the accumulation of bronchial secretions and lung infection.

The decrease in respiratory effort and muscle tone of the airways in these patients is more pronounced during sleep, with the development of obstructive sleep apnea-hypopnea syndrome (SAHS) being frequent [1, 5, 6]. Muscle weakness can also affect the heart, occasionally producing dilated cardiomyopathy, which causes heart failure and arrhythmias [7]. Also, the wasting often leads to dysphagia, swallowing disorders, and gastroesophageal reflux and, consequently, malnutrition. All of this facilitates chronic bronchial aspiration and recurrent respiratory infections in the advanced stages of NMDs [8].

Multidisciplinary care of severe and progressive NMDs has significantly evolved lately. New considerations of care are intended to better address the patients’needs; provide current advances in assessment and interventions, as well as considering emerging molecular and genetic therapies where appropriate. Currently, there has been a radical change in anticipatory diagnostic and therapeutic strategies [9]. Respiratory rehabilitation is key for addressing NMDs [5]. Two fundamental groups of rehabilitative techniques have been described, some facilitate the functioning of the diaphragm, the elimination of secretions, lung compliance, glossopharyngeal respiration and increase lung volumes [10–15], and other techniques are employed for the mobilization and prevention of musculoskeletal spasticity and stiffness [16–19].

We considered it of interest to carry out a descriptive study of patients with neuromuscular disorders, and this has revealed there exist an incidence of comorbidities and a need to prioritize rehabilitation in the groups of diseases that are expected to be greater in morbidity and mortality.

## Patients and methods

Descriptive retrospective study of pediatric and adolescent patients with neuromuscular diseases admitted to a Neuropediatric Unit at a tertiary hospital. The selected patients were divided into the three groups,these being most frequent in our setting, depending on the affected component of the motor unit: Group I:involvement of the motor neuron; Group II: peripheral neuropathies; Group III: myopathies.

### Exclusion criteria

Patients with involvement of neuromuscular junction. Likewise, the groups of the classification of genetically based neuromuscular diseases 2020 version [20].

### Inclusion criteria

NMDs patients with motor neuron involvement, with peripheral neuropathies and myopathies.

The variables that were collected in the three study groups were: demographics,associated neurological, musculoskeletal, respiratory, cardiac and digestive comorbidities, as well as the need for in-hospital respiratory and musculoskeletal physiotherapy. The criteria for prioritizing the indication for respiratory physiotherapy were: where the onset of respiratory insufficiency, as well as the presence of deformity of the chest and/or scoliosis. Depending on the clinical situation of each patient, the rehabilitation physicians of the multidisciplinary team, using the same criteria, indicated different rehabilitation methods:1) Techniques to facilitate the functioning of the diaphragm and the elimination of secretions, manual or mechanical cough assistance, slow expiration with the glottis opened in lateral decubitus position, slow expiration technique, autogenous drainage, increased expiratory flow, directed cough, forced expiration techniques, active cycle of breathing techniques,controlled inspiratory debit exercises, flow spirometer [10–14]) Techniques to mobilize and prevent musculoskeletal spasticity and stiffness: kinesitherapy and the Vojta, and Le Metayer Methods [16–18, 21, 22].

Data were obtained from the medical history of each participant after the patient’s parents or legal guardians had signed the informed consent. This study was approved by the Hospital’s Biomedical Ethics Committee and conformed tote ethical standards established in the Declaration of Helsinki of 1964. Data are expressed as mean ± SD (95% confidence intervals), median (IQR) or absolute (relative frequencies). For data that fitted a normal distribution (Shapiro-Wilk normality test) t-tests were used. Comparisons among groups were made by Kruskal-Wallis test or ANOVA. Categorical variables were assessed using the χ^2^ test or the Fisher exact test. All the tests were two-tailed, and the results were considered statistically significant when the *p* value was < 0.05. Data were analyzed with the SPSS 23.0 program (SPSS Inc., Chicago, Illinois, United States).

## Results

One hundred and three patients with NMDs admitted to the Neuropediatric Unit at our hospital were studied. Seven patients were excluded because they did not meet the inclusion criteria. From the analysis of the database, 96 patients met the inclusion criteria. The underlying pathologies presented according to the group in which patients were included were: Group I: Motor neuron involvement: Spinal Muscular Atrophy (15 patients): SMA-1: 4, of them,1 was being treated with Risdiplam, and 3 cases of SMA -1b received Nusinersen. All of them were in the sitting or standing phase. SMA-2: 11 patients. Of them, 7 received Nusinersen and 4 Risdiplam, with stabilization of their disease, measured by motor scales [23–25] and number of respiratory infections. Group II: peripheral neuropathies (40 patients): 34 chronic hereditary sensory motor neuropathies, 2 Peripheral Facial Paralysis, 1 Cranial Polyneuropathy, 1Parsonage-Turner Syndrome, 2 Guillain-Barré Syndrome. Group III: Myopathies (41 patients): 34 Muscular Dystrophies, 4 Congenital Myopathies, 2 Hipokaliemia Periodic Paralysis, 1 Mitochondrial Myopathies.

Age (years) Group I: 15 ± 9.93 years; group II: 13.33 ± 4.5 years; group III: 11.82 ± 4.9 years(p: 0.06)-. Sex (male): Group 1: 12 (80%); group II: 17 (42.5%); group III: 23 (56.1%) p: 0.03. When comparing the incidence of comorbidities in the three study groups, significance was observed in group I with involvement of the motor neuron in: Scoliosis (*p* < 0.05), Rib cage deformities (*p* < 0.001), Lung infection (p < 0.001), bronchial aspiration (*p* < 0.023), chronic respiratory insufficiency (0.001). When comparing the incidence of comorbidities in the three study groups, significance was observed in group I in: Scoliosis (*p* < 0.05), Rib cage deformities (*p* < 0.001), Lung infection (p < 0.001), bronchial aspiration (*p* < 0.023), chronic respiratory insufficiency (0.001). No significance was obtained in myocardiopathy, deglutition disorders, gastroesophageal reflux disease, sleep apnea-hypopnea syndrome and nocturnal hypoventilation. The prevalence of the digestive pathologies was higher in Group I (33.3%) when compared to Group II (10%) and III (12.2%) (Table 1).

**Table 1:** Comorbidities associated with neuromuscular diseases

	Group I. (*n* = 15)	Group II. (*n* = 40)	Group III (*n* = 41)	P
**Scoliosis**	10 (66.7%) ^a,b^	11 (27.5%)	14 (34.1%)	**0.025**
**Rib cage deformities**	4 (26.7%) ^a,b^	0	0	**< 0.001**
**Myocardiopathy**	2 (13.3) ^a,b^	0	2 (4.9%)	0.064
**Deglutition disorders y/o GERD**	5 (33.3%) ^a,b^	4 (10%)	5 (12.2%)	0.119
**Lung infection**	8 (53.3%) ^a,b^	8 (20%)	3 (7.3%)	**0.001**
**Bronchial aspiration**	2 (13.3%)	4 (10%)	0	**0.023**
**SAHS /Nocturnal hypoventilation**	3 (20%)	1 (2.5%)	2 (4.9%)	0.114
**Chronic respiratory insufficiency**	9 (60%) ^a,b^	4 (10%)	4 (9.8%)	**0.001**

Group I Involvement of the motor neuron; Group II: Peripheral neuropathies; Group III: Myopathies. *SAHS* Sleep apnea-hypopnea síndrome, *GERD* Gastroesophageal reflux disease - ^a^: *p* < 0.05 between group I and group II; ^b^: *p* < 0.05 between group I and group III

Table 2 displays the early initiation of respiratory and musculoskeletal physiotherapy, depending on the affected component of the motor unit, as well as the need for non-invasive ventilation (NIV), tracheostomy, and invasive mechanical ventilation (IMV). Group I showed significant differences with respect to the other two study groups regarding the need for early indication for respiratory physiotherapy (*p* < 0.002) and non-invasive ventilation (*p* < 0.001).

**Table 2:** Respiratory treatment and rehabilitation

	Group I. (*n* = 16)	Group II. (*n* = 41)	Group III (*n* = 42)	P
**Musculoskeletal Physiotherapy**	13 (86.7%)	30 (75%)	25 (61%)	0.117
**Respiratory physiotherapy**	11 (73.3)^a,b^	9 (22.5%)	13 (31.7%)	**0.002**
**NIV**	7 (46.7%) ^a,b^	3 (7.5%)	2 (4.9%)	**< 0.001**
**Tracheotomy**	1 (6.7%)	1 (2.5%)	1 (2.4%)	0.742
**IMV**	1 (6.7%)	1 (2.5%)	1 (2.4%)	0.759

Group I Involvement of the motor neuron; Group II: Peripheral neuropathies; Group III: Myopathies

*NIV* Non-invasive ventilation. *IMV* Invasive mechanical ventilation. ^a^: *p* < 0.05 between group I and group II; ^b^: *p* < 0.05 between group I and group III

## Discussion

In the present study of a series of children and adolescents with NMDs, it has been observed that Group I with motor neuron involvement presents a higher incidence of comorbidities. It is suggested, in the context of a multi-disciplinary approach, to initiate early rehabilitation treatment in this group of patients, which could improve its prognosis. Most NMDs are rare inherited conditions that have a relentlastly progressive course. Many worsen due to musculoskeletal, respiratory, digestive, nutritional, or heart problems. Some NMDs are associated with intellectual disability. For this reason, NMDs care is more beneficial when it is provided by a multidisciplinary team of professionals working together. The rehabilitation of patients with NMDs should include physical, daily activities performance, as well as the need for assistive devices assessments. With adequate physical, psychological and social support, patients can thus maintain a good quality of life [15, 17].

It is important to start proactive respiratory care, as the great variety of different neuromuscular diseases require careful planning of the treatment strategies in each case by the multidisciplinary team [2, 8, 22]. In our series, respiratory physiotherapy included different manual or mechanical techniques, such as cough assistance, slow expiration, increased expiratory flow, directed cough, forced expiration, active cycle of breathing, and flow spirometer. At the same time, kinesitherapy and the Vojta or the Le Metayer methods were applied to mobilize and prevent musculoskeletal spasticity and stiffness. Since our retrospective series of pediatric and adolescent patients with neuromuscular diseases was classified into three different pathology groups, depending on the affected component of the motor unit, to perform the same type of respiratory and musculoskeletal physiotherapy in all patients was not possible,as its indication depends on the patients’ clinical situation and age. The main objective of this work was to study the incidence of comorbidities in each group to benefit from the therapeutic resources in physiotherapy and rehabilitation, and to approach to the most serious patients with these, which should be standardized.

NMDs patients with respiratory dysfunction require rehabilitation before performing surgery [11]. In cases without respiratory dysfunction, early physiotherapy is not required; however, surgical correction can trigger exacerbation and respiratory insufficiency. Respiratory dysfunction can manifest with dyspnea, worsening fatigue, an increased frequency of lower respiratory tract infections, and diaphoresis. Signs of nocturnal hypoventilation are manifested by agitation during sleep, headache, or nausea upon awakening, as well as drowsiness during the day and decreased concentration. By means of pulmonary function tests and polysomnography, an objective evaluation of daytime and/or nocturnal chronic alveolar hypoventilation can be made. Depending on the results of these tests, different physiotherapy techniques are indicated considering age and pathology. Where necessary, non-invasive ventilation (NIV) is indicated at night and/or during the day. On the other hand, inpatients at high risk of respiratory insufficiency, the possible need for tracheostomy should be considered and, depending on the patient’s clinical situation, IMV should be indicated to improve lung expansion [19].

When addressing NMDs, we considered, as suggested by Estrup et al. [26], the training of the respiratory muscles, which could improve vital capacity and resistance to muscle fatigue. In neuromuscular diseases, respiratory physiotherapy is of vital importance, as it facilitates the maintenance of lung function and reduces the incidence of lung infections [2]. In our study, despite respiratory physiotherapy was performed more intensively in Group I with motor neuron involvement than in Group II and III, this group presented a greater number of complications, that is, more cases required NIV, tracheostomy and IMV. This was to be expected, since the incidence of respiratory disease in Group I was higher. Preventive indication for early respiratory physiotherapy in these patients could improve lung expansion and volume and prevent a decline in the respiratory function.

It is important to consider that children with NMDs also have secondary osteoporosis. Low bone mass in these patients causes greater bone fragility and risk of fracture [27, 28]. In our study, Group I showed the highest prevalence of musculoskeletal pathology and digestive disorders, as other authors have as wellreported [29–31]. Weakness of the intercostal muscles, scoliosis, respiratory insufficiency, and infections are the main complications that Group I patients presented.

Regarding musculoskeletal physiotherapy, as well in Group I, it is more common to find contractures in the limbs, musculoskeletal spasticity, and rigidity [16, 18]. There is controversy among authors in relation to the approach to neuromuscular diseases, sleep disorders, as well as reducing the incidence of respiratory pathology. Chatwin et al. [6] have suggested the use of NIV; however, Marques et al. [31]  and Toussaint et al. [32] have advised the use of a self inflating bag to perform hyperinflation and thus increase the maximum peak of assisted and unassisted cough. In our series, we utilized NIV routinely as respiratory support. Concerning Group II with peripheral neuropathies, the prevalence of comorbidities was lower than in Group I. On the other hand, the incidence of lung infection and bronchial aspiration was higher in Group II than in Group III, although without significance. As described by Pons et al. [33], Group II patients frequently present phrenic nerve paralysis, which per se causes respiratory insufficiency. In order to address patients in this group, Darquennes et al. [34] have recommended the use of nocturnal NIV to treat respiratory disease, as with Group I. We followed a similar pattern. Group III also presented a lower prevalence of comorbidities than Group I. We observed a higher incidence of scoliosis in Group III than in Group II. Consequently, neuromuscular diseases with motor disturbance present more comorbidities in children and adolescents, which is due to central affectation. On the contrary, in Groups II and III with neuropathies and myopathies, the injury is more peripheral and localized.

The present study is of interest since, based on our results, we suggest a large multicenter prospective study to know the incidence of comorbidities in NMDs and to prioritize an approach to these. The major strength of this study is that it was conducted in a single center with minimal variability in data collection, establishing restrictive and objective inclusion criteria.

When studying neuromuscular diseases in children and adolescents in our environment, we observed that, in Group I with motor neuron disturbance, there was also a greater musculoskeletal involvement, rib cage deformity and scoliosis, as well as a higher prevalence of lung infection, bronchial aspiration, and a greater need for NIV. Respiratory and musculoskeletal physiotherapeutic treatment was indicated more intensively in Group I patients. The patients with motor neuron involvement, as they presented more comorbidities, required a narrower multidisciplinary approach, in which early rehabilitative treatment, from the onset of diagnosis, was key to improve their prognosis.

## Data Availability

The datasets used are available from DLTA on reasonable request.
